# Ferrovalley and Quantum Anomalous Hall Effect in Janus TiTeCl Monolayer

**DOI:** 10.3390/ma17133331

**Published:** 2024-07-05

**Authors:** Yufang Chang, Zhijun Zhang, Li Deng, Yanzhao Wu, Xianmin Zhang

**Affiliations:** 1 Public Basic Department, Shenyang Conservatory of Music, Shenyang 110818, China; changyf537@foxmail.com; 2 School of Electrical and Automation Engineering, Liaoning Institute of Science and Technology, Benxi 117004, China; zhangzhijun@lnist.edu.cn; 3 School of Material Science and Engineering, Northeastern University, Shenyang 110819, China; 2210162@stu.neu.edu.cn (L.D.); 2010235@stu.neu.edu.cn (Y.W.)

**Keywords:** first-principles calculations, compressive strain, quantum anomalous hall effect, ferrovalley

## Abstract

Ferrovalley materials are garnering significant interest for their potential roles in advancing information processing and enhancing data storage capabilities. This study utilizes first-principles calculations to determine that the Janus monolayer TiTeCl exhibits the properties of a ferrovalley semiconductor. This material demonstrates valley polarization with a notable valley splitting of 80 meV. Additionally, the Berry curvature has been computed across the first Brillouin zone of the monolayer TiTeCl. The research also highlights that topological phase transitions ranging from ferrovalley and half-valley metals to quantum anomalous Hall effect states can occur in monolayer TiTeCl under compressive strains ranging from −1% to 0%. Throughout these strain changes, monolayer TiTeCl maintains its ferromagnetic coupling. These characteristics make monolayer TiTeCl a promising candidate for the development of new valleytronic and topological devices.

## 1. Introduction

Valleytronics has emerged as an appealing approach to information encoding, propelled by recent advances in two-dimensional (2D) materials. This field leverages a unique valley degree of freedom, in addition to the traditional charge and spin, offering a robust and effective mechanism for information processing in future nanoscale electronic devices [1,2,3,4,5,6,7]. In this context, a valley refers to local maxima or minima in the material’s conduction or valence bands. Such materials feature valleys that are degenerate yet distinct at different k points in momentum space. Achieving valley splitting, which is essential for practical valleytronic devices, requires overcoming their inherent degeneracy [8,9,10,11]. The various methods used to induce valley splitting in nonmagnetic materials include magnetic doping [12], optical pumping [13,14], magnetic proximity effects [15,16], and the application of static magnetic fields [17]. However, maintaining valley band structures after material growth through these techniques is still challenging in experiments [18,19,20,21]. The introduction of two-dimensional ferrovalley (FV) materials, which combine ferromagnetic (FM) and valley characteristics, hopefully addresses these issues [22,23].

Among the diverse 2D FV materials, significant attention has been paid to Janus structures such as the LaBrI monolayer [24], FeClBr monolayer [18], VSSe monolayer [25,26], and VSeTe monolayer [27,28]. These materials typically combine halogens or sulfur with transition metals to form stable layers. Additionally, innovative configurations using both sulfur and halogens as anions, with transition metals as cations, have led to the development of other 2D FV materials like the CrXY, VSCl, CrSCl, and TiXY monolayers [29,30,31,32]. These materials are named Janus materials because of their different chemical compositions on the upper and lower sides of transition metals’ cations [33]. Recent explorations into TiTeI and TiTeBr monolayers have also highlighted their potential as 2D FV candidates [34,35]. These materials exhibit a valley-dependent Berry curvature under an in-plane electric field, leading to the anomalous valley Hall effect, which is amazing for advanced applications [36,37,38,39].

In this research, the Janus monolayer TiTeCl has been identified as an FM semiconductor through first-principles calculations. It displays valley polarization with an 80 meV valley splitting. The Berry curvature is computed across the first Brillouin zone for TiTeCl. Additionally, this study observes topological phase transitions including FV, half-valley metal (HVM), and quantum anomalous Hall (QAH) effects in TiTeCl when it is subjected to compressive strains ranging from −1% to 0%. The material retains its FV properties under compressive strains stretching from 0 to −0.63%. The transformation from FV to HVM occurs under strains of −0.63% and −0.83%. Within this strain interval, a QAH phase emerges, reverting to FV characteristics when the strain exceeds −0.83%. This investigation proposes monolayer TiTeCl as a versatile material for probing the dynamics of valleys, spin, and topological phenomena.

## 2. Computational Method and Details

Density functional theory (DFT) calculations were carried out using the Vienna ab initio simulation package [40,41]. Ion–electron interactions were modeled with the projector augmented wave (PAW) pseudopotential [42], and the exchange–correlation potentials were described by the Perdew–Burke–Ernzerhof (PBE) formulation of the generalized gradient approximation (GGA) [43]. To address the strong correlation effects in Ti-3d electrons, we employed the PBE+U method, setting U to 2.8 eV [44,45,46,47]. We chose a plane wave base set with a cut-off energy of 500 eV and sampled the first Brillouin zone (BZ) integral using a Γ-centered 17 × 17 × 1 Monkhorst-pack k-point grid [48]. To prevent artificial interactions between periodic images, we added a vacuum space of 20 Å along the z/c direction. All atoms were fully relaxed until the Hermann–Feynman forces were below 0.01 eV/Å, with an energy convergence criterion of 10^−6^ eV. Phonon dispersion calculations used a 3 × 3 × 1 supercell in the PHONOPY package [49,50]. Ab initio molecular dynamics (AIMD) simulations were conducted at 300 K using an NVT ensemble [51]. Monte Carlo simulations were carried out with the mcsolver package [52,53], and calculation data were processed via the VASPKIT package [54]. We also developed a maximized localization function using the WANNIER90 package and performed post-processing with the WANNIERTOOLS software [55,56]

## 3. Results and Discussion

**Figure 1:**
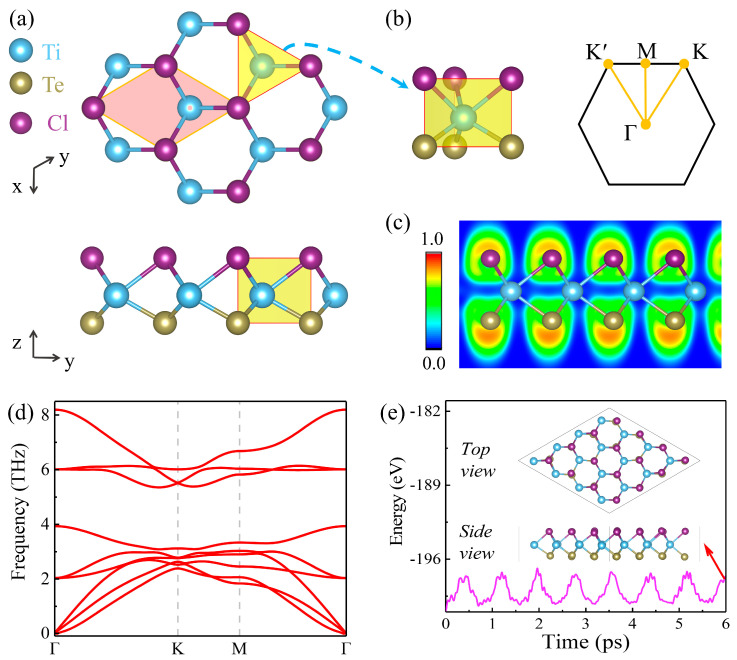
(**a**) Overview of Janus monolayer TiTeCl that depicts the crystal structure, highlighting the unit cell with a red overlay. (**b**) The trigonal prismatic geometry (**left**) alongside the first Brillouin zone marked with high-symmetry points (**right**). (**c**) The electron localization function. (**d**) The phonon spectrum. (**e**) The total energy variation in an AIMD simulation, with the insets featuring a structure snapshot after 6 ps at 300 K.

Figure 1a illustrates the hexagonal structure of the Janus TiTeCl monolayer, categorized under the P3m1 space group. The titanium (Ti) atoms form the core layer, flanked by chlorine (Cl) atoms on top and tellurium (Te) atoms at the bottom. The coordination, depicted on the left in Figure 1b, involves each Ti atom bonding with three atoms of both Te and Cl, thereby disrupting inversion symmetry [13,57,58,59]. This configuration also features in the Brillouin zone, shown on the right of Figure 1b, aligning with the monolayer’s hexagonal layout. Figure 1c demonstrates that electron localization is predominantly around the Te and Cl atoms, with minimal electron presence elsewhere, showcasing the ionic nature of the Ti-Te/Cl bonds. Stability assessments through phonon spectrum analysis and ab initio molecular dynamics (AIMD) simulations confirm the material’s dynamical and thermal stability, respectively. The phonon spectrum lacks imaginary frequencies (Figure 1c), while the AIMD simulations, over 6 ps, show consistent total energy and no structural distortions (Figure 1d). The optimized lattice constant for the TiTeCl monolayer is recorded at 3.86 Å.

**Figure 2:**
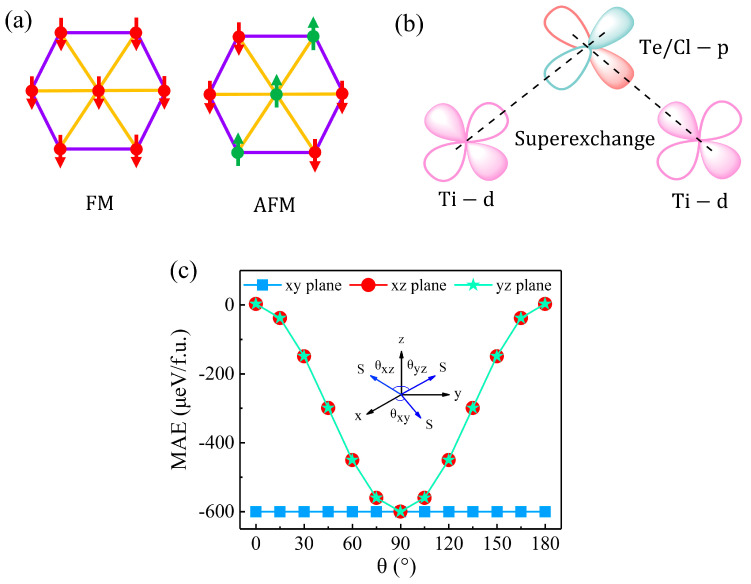
(**a**) Illustration of both FM and AFM states in a TiTeCl monolayer. (**b**) Diagram depicting the superexchange mechanism between Ti and Te atoms through I-Ti, mediated by d-p-d orbital interactions. (**c**) The variation of the MAE as a function of the angle θ in the xy, xz, and yz planes of the TiTeCl monolayer. The accompanying inset graphically represents the rotation of the spin vector S from 0 to 180° in each plane.

The magnetic characteristics of the TiTeCl monolayer have been investigated, focusing on its magnetic base state through a comparison of FM and AFM configurations, as illustrated in Figure 2a. A significant energy difference of 99.62 meV/f.u. between these states indicates a preference for the FM state in TiTeCl. The monolayer’s magnetic moment per unit cell is 1 μ_B_. Notably, the bond angles for Ti-Te-Ti and Ti-Cl-Ti are 81.97° and 86.57°, respectively, approaching 90°. These configurations support their FM coupling preference according to the Goodenough–Kanamori–Anderson (GKA) rules [60], as depicted in Figure 2b.

Magnetic anisotropy energy (MAE) is essential for establishing long-range magnetic order in two-dimensional (2D) magnetic systems. MAE, influenced by spin–orbit coupling (SOC), is calculated by the difference in total energies when magnetic moments align in different directions, specifically MAE = E_z_ − E_θ_. Here, E_z_ and E_θ_ represent the system’s total energies when magnetic moments are directed along the [001] axis and an angle θ in the plane, as depicted in the inset of Figure 2c. The MAE changes between −603 µeV/f.u. and 0 µeV/f.u. across the xz and yz planes, remaining constant at 0 µeV/f.u. in the xy plane, which indicates an in-plane easy axis for the monolayer TiTeCl with a minimum MAE value of −603 µeV/f.u.

**Figure 3:**
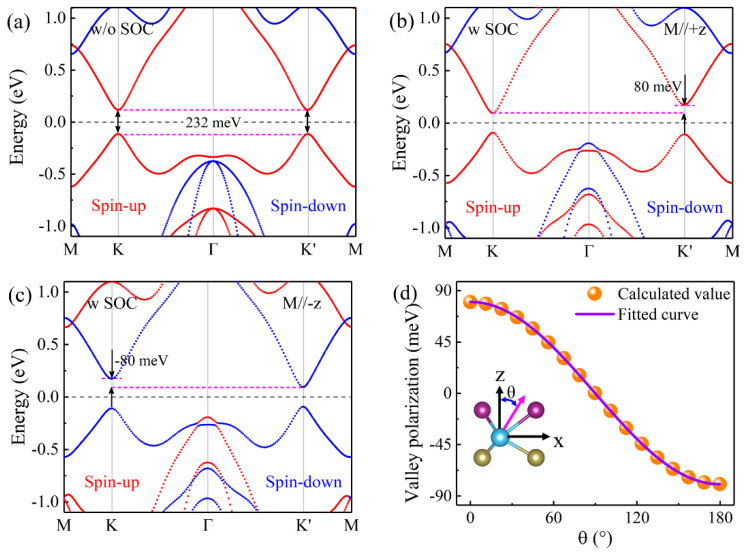
(**a**) Spin-resolved energy band structure of monolayer TiTeCl without a SOC effect. Spin-resolved energy band structure of monolayer TiTeCl with SOC effect as the magnetization of the Ti atom along the (**b**) +z and (**c**) −z directions, respectively. (**d**) Valley polarization of monolayer TiTeCl with different polar angles of spin orientation.

The electronic properties of the TiTeCl monolayer have been analyzed through calculations, revealing its structure in the absence and presence of spin–orbit coupling (SOC). As depicted in Figure 3a, without a SOC (w/o SOC) effect, the TiTeCl monolayer exhibits FM semiconductor behavior, characterized by a direct band gap of 232 meV. The electron and hole bands nearest the Fermi level are predominantly influenced by identical spins. An intriguing feature is the appearance of degenerate Dirac valleys at the K and Kʹ points, just near the Fermi level. Incorporating SOC (w SOC) effects, as shown in Figure 3b, disrupts this degeneracy due to the magnetization of Ti atoms aligned along the +z direction, resulting in a valley splitting of 80 meV in the conduction band, characteristic of FV behavior. This pattern mirrors the findings in TiSCl and TiSeBr monolayers [29]. Reversing the magnetization direction to −z also inversely modifies the valley polarization while maintaining the magnitude of the valley splits, as illustrated in Figure 3c. Notably, the valley polarization in the valence band is very small, which can be attributed to the contributions of specific orbitals to the band edges [18,22,39], as indicated in Figure S1. Moreover, the dependences of valley polarization on the polar angle (θ) of the spin orientation for the TiTeCl monolayer are studied, as shown in Figure 3d. It is found that the data can be well fitted by a cosine function, which is consistent with our research results in Figure S1. This phenomenon is similar to what has been observed in FeClBr [18], VSe_2_ [22], and VSi_2_N_4_ [39] monolayers. This indicates that the valley freedom can be regulated by controlling the magnetization direction of the Ti atom through an external magnetic field.

Analogous to the H phase of monolayer MoS_2_ [61], monolayer TiTeCl is expected to demonstrate circular dichroism, characterized by its degree of optical polarization, denoted as η(k) [62,63].

(1) $$\eta (k)=\frac{{\left|P_{+}^{cv}(k)\right|}^{2}-{\left|P_{-}^{cv}(k)\right|}^{2}}{{\left|P_{+}^{cv}(k)\right|}^{2}+{\left|P_{-}^{cv}(k)\right|}^{2}}$$

**Figure 4:**
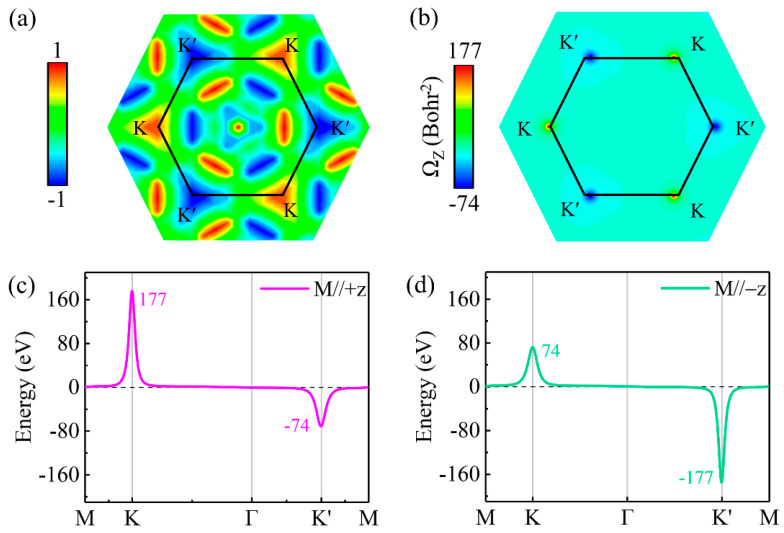
(**a**) The degree of circular polarization between the valence and conduction bands in a monolayer of TiTeCl. (**b**) The Berry curvature across the first Brillouin zone. The Berry curvature along the high-symmetry line with the Ti atom’s magnetization oriented in the (**c**) +z and (**d**) −z directions, respectively.

The circular polarization transition matrix elements, denoted by $P_{\pm }^{cv}(k)=\frac{1}{\sqrt{2}}(P_{x}^{cv}(k)\pm iP_{y}^{cv}(k))$, characterize transitions involving circularly polarized light. These elements are derived from the interband matrix component $P^{cv}(k)=\langle \psi_{ck}\left|\hat{p}\right|\psi_{vk}\rangle$, which can be computed using DFT methods. Illustrated in Figure 4a, the optical polarization values at the K and K′ valleys are exactly +1.0 and −1.0, respectively, indicating that photons with right-handed and left-handed circular polarization are preferentially absorbed at these respective valleys.

Simultaneously, the disruption of inversion symmetry leads to divergent Berry curvatures at the K and K’ valleys, as derived using the Kubo formula [64,65]:

(2) $$\Omega_{z}\left(k\right)=-\sum_{n}\sum_{n\neq m}f_{n}\left(k\right)\frac{2Im\langle \psi_{nk}\left|{\hat{v}}_{x}\right|\psi_{mk}\rangle \langle \psi_{mk}\left|{\hat{v}}_{y}\right|\psi_{nk}\rangle }{{\left(E_{nk}-E_{mk}\right)}^{2}}$$

The Fermi–Dirac distribution, denoted by $f_{n}\left(k\right)$, where *k* represents the electron wave vector, plays a crucial role in determining the behavior of electrons. The component ${\hat{v}}_{x}({\hat{v}}_{y})$ is the x (y)-direction velocity operator and $E_{nk}(E_{mk})$ refers to the Bloch wave function eigenvalues of $\psi_{nk}\;(\psi_{mk})$. As illustrated in Figure 4b–d, the Berry curvature across the Brillouin zone (BZ) and along high-symmetry lines is examined. From Figure 4b, it is evident that, at the K and K’ points, the Berry curvature values, $\Omega_{z}\left(k\right)$, are 177 and −74 Bohr^2^ respectively, highlighting the distinct valley characteristics in a monolayer of TiTeCl. The linear representation of Berry curvature in Figure 4c provides a clearer depiction of these valley contrasts. Importantly, a reversal in magnetization direction swaps the values between the two valleys without altering their signs, as Figure 4d shows. With a longitudinal in-plane electric field *E*, the non-zero out-of-plane $\Omega_{z}\left(k\right)$ imparts an unusual lateral velocity to the electron carriers, $v_{⊥}=-\frac{e}{\hbar }E\times \Omega_{z}\left(k\right)$, demonstrating the anomalous valley Hall effect in monolayer TiTeCl [14,65].

**Figure 5:**
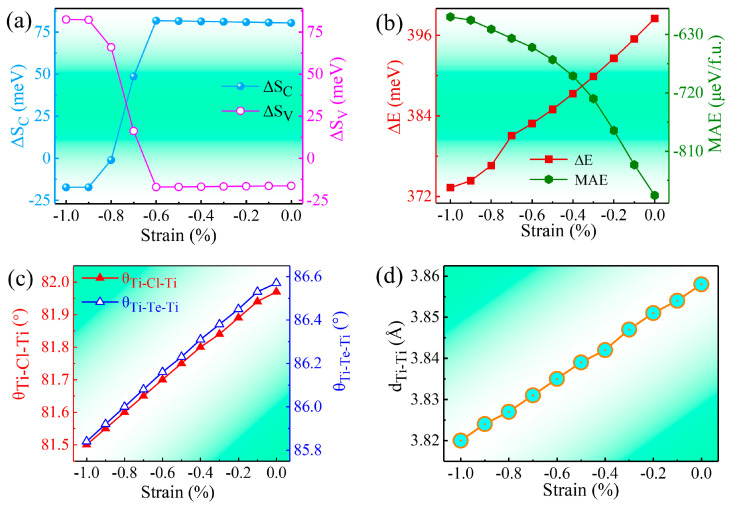
Effects of biaxial strains on monolayer TiTeCl properties: (**a**) compares the valley splitting at the conduction band minimum (ΔS_C_), with that at the valence band maximum (ΔS_V_). (**b**) details the variations in energy difference (ΔE) and magnetic anisotropy energy (MAE). (**c**) illustrates the adjustments in the angles between the Ti-Te-Ti and Ti-Cl-Ti bonds. (**d**) demonstrates the modifications in the spacing between adjacent Ti atoms as strain varies.

Strain profoundly affects the physical characteristics of 2D FV materials [30,34,38]. In this context, the influence of biaxial strains ranging from −1% to 0% on the valley polarization and magnetic attributes of TiTeCl monolayers is examined. The strain parameter, η, is calculated using the formula $\eta =(\alpha -\alpha_{0})/\alpha$, where $\alpha_{0}$ is the lattice constant of the unstrained TiTeCl monolayer and α represents the lattice constant under strain. A negative η value indicates compressive strain. In the strain range of −1% to 0%, Figure 5a displays the valley splitting of both the conduction band minimum (ΔS_C_) and valence band maximum (ΔS_V_) of the TiTeCl monolayer. In the range of 0~−0.7% strain, valley splitting predominantly arises from the conduction band minimum (CBM). With increasing compressive strain over −0.7%, the contribution from the CBM decreases significantly, whereas the valley splitting from the valence band maximum (VBM) increases sharply, becoming the dominant factor at strains of −0.8%, −0.9%, and −1.0%. Similar behavior under comparable strains is observed in other FM van der Waals materials such as (Ru, Os)Br_2_ [66], FeCl_2_ [38,67], and VSi_2_(N, P)_4_ [68,69]. During these strain adjustments, the monolayer maintains FM coupling and an in-plane easy magnetic axis, as depicted in Figure 5b. Furthermore, the angles and distances in the Ti-Te-Ti and Ti-Cl-Ti bonds, as well as between adjacent Ti atoms, are reduced as compressive strain increases, as shown in Figure 5c,d, respectively.

**Figure 6:**
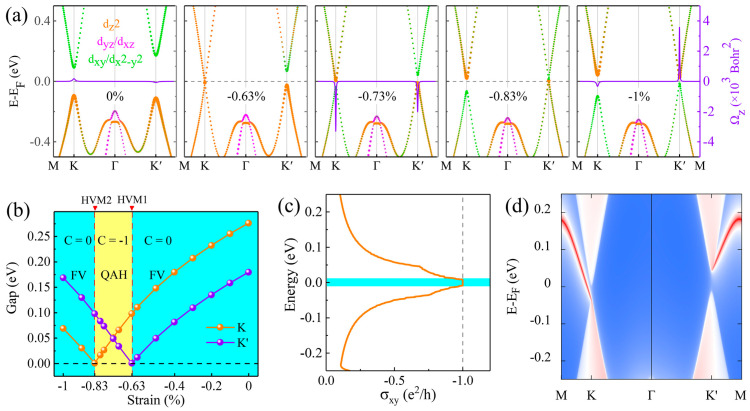
Characteristics of monolayer TiTeCl under varying strains. (**a**) displays the orbital-resolved energy band structures incorporating the effects of SOC and linear Berry curvatures along high-symmetry points. (**b**) illustrates the band gaps at the K and K’ valleys along with a phase diagram highlighting the transitions between the FV and QAH phases, marked by two HVM states indicated with red arrows. (**c**) shows the anomalous Hall conductivity. (**d**) examines the chiral edge states at −0.73% strain in monolayer TiTeCl.

Figure 6a illustrates the impact of varying compressive strains on the orbital-specific energy band structures in a TiTeCl monolayer. Initially, unstrained TiTeCl is characterized as an FV semiconductor with CBM and VBM primarily arising from $d_{xy}$/$d_{x^{2}-y^{2}}$ and $d_{z^{2}}$ orbitals, respectively. At a compressive strain of −0.63%, the CBM and VBM converge at the K point, transitioning to a HVM1 state. Increasing the strain to −0.73%, the CBM consists of the $d_{z^{2}}$ orbital, whereas the VBM originates from the $d_{xy}$/$d_{x^{2}-y^{2}}$ orbital at the K’ point, indicating a band inversion that imparts a QAH characteristic to the TiTeCl monolayer.

This strain level also alters the Berry curvature at the K point from positive to negative. At −0.83%, the CBM and VBM meet again at the K’ point, shown as the HVM2 state. Upon further strain, the monolayer reverts to its semiconductor state by −1% strain, with a reversed band and Berry curvature at both K and K’ points, as depicted in Figure 6a. Figure 6b delineates the variations in band gaps at the K and K’ valleys and the phase diagram for different strains. Additionally, to substantiate the QAH property, the anomalous Hall conductivity and edge states at −0.73% strain have been calculated with the following equation [70,71]:

(3) $$\sigma_{xy}=-\frac{e^{2}}{\hbar }\int_{BZ}\frac{dk}{{\left(2\pi \right)}^{2}}\Omega_{z}\left(k\right)$$

Figure 6c displays that the anomalous Hall conductivity of monolayer TiTeCl is quantized at −1.0 e^2^/h, confirming a Chern number of −1. This property is underscored by the presence of a chiral edge state bridging the valence and conduction bands, as shown in Figure 6d. Notably, from an experimental perspective, the present TiTeCl monolayer may be easily grown by spin coating [72,73] or chemical vapor deposition [74] methods.

## 4. Conclusions

This research on the characteristics of monolayer TiTeCl has revealed its stability and various functional properties. This material is identified as an FM semiconductor featuring a direct band gap of 232 meV. It also displays in-plane magnetic anisotropy, with a MAE of 603 μeV/f.u. Notably, monolayer TiTeCl exhibits a valley polarization, characterized by a valley splitting of approximately 80 meV. Under compressive strains ranging from 0% to −0.63%, the Janus structure of monolayer TiTeCl maintains its FV behavior. The transition to an HVM state occurs when the strain is adjusted between −0.63% and −0.83%. Within this strain interval, a QAH phase emerges, yet the FV properties reappear when the strain exceeds −0.83%. These dynamic phase changes highlight the potential of monolayer TiTeCl for application in advanced valleytronics.

## Data Availability

The original contributions presented in the study are included in the article/Supplementary Material, further inquiries can be directed to the corresponding author.

## Supplementary Materials

The following supporting information can be downloaded at: https://www.mdpi.com/article/10.3390/ma17133331/s1, Figure S1: Projected band structures of Ti-d orbitals for TiTeCl monolayer [18,22,39].

## References

[1] Schaibley J.R., Yu H., Clark G., Rivera P., Ross J.S., Seyler K.L., Yao W., Xu X. (2016). Valleytronics in 2D materials. Nat. Rev. Mater..

[2] Mak K.F., Xiao D., Shan J. (2018). Light–valley interactions in 2D semiconductors. Nat. Photonics.

[3] Neto A.H.C., Guinea F., Peres N.M.R., Novoselov K.S., Geim A.K. (2009). The electronic properties of graphene. Rev. Mod. Phys..

[4] Vitale S.A., Nezich D., Varghese J.O., Kim P., Gedik N., Jarillo-Herrero P., Xiao D., Rothschild M. (2018). Valleytronics: Opportunities, challenges, and paths forward. Small.

[5] Shimazaki Y., Yamamoto M., Borzenets I.V., Watanabe K., Taniguchi T., Tarucha S. (2015). Generation and detection of pure valley current by electrically induced Berry curvature in bilayer graphene. Nat. Phys..

[6] Wu Y., Tong J., Deng L., Luo F., Tian F., Qin G., Zhang X. (2023). Coexisting ferroelectric and ferrovalley polarizations in bilayer stacked magnetic semiconductors. Nano Lett..

[7] Wu Y., Tong J., Deng L., Luo F., Tian F., Qin G., Zhang X. (2023). Realizing spontaneous valley polarization and topological phase transitions in monolayer ScX_2_ (X = Cl, Br, I). Acta Mater..

[8] Wu Y., Deng L., Tong J., Yin X., Tian F., Qin G., Zhang X. (2023). Ferrovalley and topological phase transition behavior in monolayer Ru(OH)_2_. J. Mater. Chem. C.

[9] Zhang Y.B., Tan Y.W., Stormer H.L., Kim P. (2005). Experimental observation of the quantum Hall effect and Berry’s phase in graphene. Nature.

[10] Liu Y., Gao Y., Zhang S., He J., Yu J., Liu Z. (2019). Valleytronics in transition metal dichalcogenides materials. Nano Res..

[11] Zhao S., Li X., Dong B., Wang H., Wang H., Zhang Y., Han Z., Zhang H. (2021). Valley manipulation in monolayer transition metal dichalcogenides and their hybrid systems: Status and challenges. Rep. Prog. Phys..

[12] Peng R., Ma Y., Zhang S., Huang B., Dai Y. (2018). Valley polarization in Janus single-layer MoSSe via magnetic doping. J. Phys. Chem. Lett..

[13] Zeng H., Dai J., Yao W., Xiao D., Cui X. (2012). Valley polarization in MoS_2_ monolayers by optical pumping. Nat. Nanotechnol..

[14] Hsu W.-T., Chen Y.-L., Chen C.-H., Liu P.-S., Hou T.-H., Li L.-J., Chang W.-H. (2015). Optically initialized robust valley-polarized holes in monolayer WSe_2_. Nat. Commun..

[15] Ma Y., Wu Y., Tong J., Deng L., Yin X., Zhou L., Han X., Tian F., Zhang X. (2023). Distinct ferrovalley characteristics of the Janus RuClX (X = F, Br) monolayer. Nanoscale.

[16] Li N., Zhang J., Xue Y., Zhou T., Yang Z. (2018). Large valley polarization in monolayer MoTe_2_ on a magnetic substrate. Phys. Chem. Chem. Phys..

[17] Smoleński T., Goryca M., Koperski M., Faugeras C., Kazimierczuk T., Bogucki A., Nogajewski K., Kossacki P., Potemski M. (2016). Tuning valley polarization in a WSe_2_ monolayer with a tiny magnetic field. Phys. Rev. X.

[18] Li R., Jiang J., Mi W., Bai H. (2021). Room temperature spontaneous valley polarization in two-dimensional FeClBr monolayer. Nanoscale.

[19] Sygletou M., Tzourmpakis P., Petridis C., Konios D., Fotakis C., Kymakis E., Stratakis E. (2016). Laser induced nucleation of plasmonic nanoparticles on two-dimensional nanosheets for organic photovoltaics. J. Mater. Chem. A.

[20] Tsikritzis D., Rogdakis K., Chatzimanolis K., Petrovic M., Tzoganakis N., Najafi L., Martin-Garcia B., Oropesa-Nunez R., Bellani S., Castillo A. (2020). A two-fold engineering approach based on Bi_2_Te_3_ flakes towards efficient and stable inverted perovskite solar cells. Mater. Adv..

[21] Tsikritzis D., Chatzimanolis K., Bellani N.T.S., Zappia M.I., Bianca G., Curreli N., Buha J., Kriegel I., Antonatos N., Sofer Z. (2022). Two-dimensional BiTeI as a novel perovskite additive for printable perovskite solar cells. Sustain. Energy Fuels.

[22] Tong W.-Y., Gong S.-J., Wan X., Duan C.-G. (2016). Concepts of ferrovalley material and anomalous valley Hall effect. Nat. Commun..

[23] Zhang L., Yang Z., Gong T., Pan R., Wang H., Guo Z., Zhang H., Fu X. (2020). Recent advances in emerging Janus two-dimensional materials: From fundamental physics to device applications. J. Mater. Chem. A.

[24] Jiang P., Kang L., Li Y.-L., Zheng X., Zeng Z., Sanvito S. (2021). Prediction of the two-dimensional Janus ferrovalley material LaBrI. Phys. Rev. B.

[25] Zhang C., Nie Y., Sanvito S., Du A. (2019). First-principles prediction of a room-temperature ferromagnetic Janus VSSe monolayer with piezoelectricity, ferroelasticity, and large valley polarization. Nano Lett..

[26] Luo C., Peng X., Qu J., Zhong J. (2020). Valley degree of freedom in ferromagnetic Janus monolayer H-VSSe and the asymmetry-based tuning of the valleytronic properties. Phys. Rev. B.

[27] Guan Z., Ni S. (2020). Predicted 2D ferromagnetic Janus VSeTe monolayer with high Curie temperature, large valley polarization and magnetic crystal anisotropy. Nanoscale.

[28] Guan Z., Ni S. (2020). Strain-controllable high Curie temperature, large valley polarization, and magnetic crystal anisotropy in a 2D ferromagnetic Janus VSeTe monolayer. ACS Appl. Mater. Interfaces.

[29] Hou Y., Xue F., Qiu L., Wang Z., Wu R. (2022). Multifunctional two-dimensional van der Waals Janus magnet Cr-based dichalcogenide halides. NPJ Comput. Mater..

[30] Yang H., Song M., Li Y., Guo Y., Han K. (2022). Ferromagnetism and valley polarization in Janus single-layer VSCl. Phys. E Low-Dimens. Syst. Nanostruct..

[31] Guo S.-D., Guo X.-S., Zhu Y.-T., Ang Y.-S. (2022). Predicted ferromagnetic monolayer CrSCl with large vertical piezoelectric response: A first-principles study. Appl. Phys. Lett..

[32] Sheng K., Yuan H.-K., Zhang B. (2022). Intrinsic spin, valley and piezoelectric polarizations in room-temperature ferrovalley Janus TiXY (XY = SCl and SeBr) monolayers. Nanoscale.

[33] Kiseleva N., Filatov M.A., Oldenburg M., Busko D., Jakoby M., Howard I.A., Richards B.S., Senge M.O., Borisov S.M., Turshatov A. (2018). The Janus-faced chromophore: A donor–acceptor dyad with dual performance in photon up-conversion, Chemical Communications. Chem. Commun..

[34] Ji S., Yao R., Quan C., Yang J., Caruso F., Li X. (2023). Anomalous valley Hall effect induced by mirror symmetry breaking in transition metal dichalcogenides. Phys. Rev. B.

[35] Su B., Peng X., Yan Z., Lin L., Huang X., Liu J. (2024). Large valley polarization and the valley-dependent Hall effect in a Janus TiTeBr monolayer. Phys. Chem. Chem. Phys..

[36] Sun H., Li S.-S., Ji W.-X., Zhang C.-W. (2022). Valley-dependent topological phase transition and quantum anomalous valley Hall effect in single-layer RuClBr. Phys. Rev. B.

[37] Liu P., Liu S., Jia M., Yin H., Zhang G., Ren F., Wang B., Liu C. (2022). Strain-driven valley states and phase transitions in Janus VSiGeN_4_ monolayer. Appl. Phys. Lett..

[38] Sheng K., Zhang B., Yuan H.-K., Wang Z.-Y. (2022). Strain-engineered topological phase transitions in ferrovalley 2H-RuCl_2_ monolayer. Phys. Rev. B.

[39] Cui Q., Zhu Y., Liang J., Cui P., Yang H. (2021). Spin-valley coupling in a two-dimensional VSi_2_N_4_ monolayer. Phys. Rev. B.

[40] Kresse G., Furthmüller J. (1996). Efficient iterative schemes for ab initio total-energy calculations using a plane-wave basis set. Phys. Rev. B.

[41] Kresse G., Furthmüller J. (1996). Efficiency of ab-initio total energy calculations for metals and semiconductors using a plane-wave basis set. Comput. Mater. Sci..

[42] Blöchl P.E. (1994). Projector augmented-wave method. Phys. Rev. B.

[43] Perdew J.P., Burke K., Ernzerhof M. (1996). Generalized gradient approximation made simple. Phys. Rev. Lett..

[44] Liechtenstein A.I., Anisimov V.I., Zaanen J. (1995). Density-functional theory and strong interactions: Orbital ordering in Mott-Hubbard insulators. Phys. Rev. B.

[45] Nolan M., Elliott S.D., Mulley J.S., Bennett R.A., Basham M., Mulheran P. (2008). Electronic structure of point defects in controlled self-doping of the TiO_2_ (110) surface: Combined photoemission spectroscopy and density functional theory study. Phys. Rev. B.

[46] Poteryaev A.I., Lichtenstein A.I., Kotliar G. (2004). Nonlocal Coulomb interactions and metal-insulator transition in Ti_2_O_3_: A cluster LDA + DMFT approach. Phys. Rev. Lett..

[47] Cuong D.D., Lee B., Choi K.M., Ahn H.-S., Han S., Lee J. (2007). Oxygen vacancy clustering and electron localization in oxygen-deficient SrTiO_3_ LDA + U Study. Phys. Rev. Lett..

[48] Monkhorst H.J., Pack J.D. (1976). Special points for Brillouin-zone integrations. Phys. Rev. B.

[49] Baroni S., De Gironcoli S., Corso A.D., Giannozzi P. (2001). Phonons and related crystal properties from density-functional perturbation theory. Rev. Mod. Phys..

[50] Togo A., Tanaka I. (2015). First principles phonon calculations in materials science. Scr. Mater..

[51] Martyna G.J., Klein M.L., Tuckerman M. (1992). Nosé–Hoover chains: The canonical ensemble via continuous dynamics. J. Chem. Phys..

[52] Liu L., Ren X., Xie J., Cheng B., Liu W., An T., Qin H., Hu J. (2019). Magnetic switches via electric field in BN nanoribbons. Appl. Surf. Sci..

[53] Huang C., Feng J., Zhou J., Xiang H., Deng K., Kan E. (2019). Ultra-high-temperature ferromagnetism in intrinsic tetrahedral semiconductors. J. Am. Chem. Soc..

[54] Wang V., Xu N., Liu J.-C., Tang G., Geng W.-T. (2021). VASPKIT: A user-friendly interface facilitating high-throughput computing and analysis using VASP code. Comput. Phys. Commun..

[55] Mostofi A.A., Yates J.R., Pizzi G., Lee Y.-S., Souza I., Vanderbilt D., Marzari N. (2014). An updated version of wannier90: A tool for obtaining maximally-localised Wannier functions. Comput. Phys. Commun..

[56] Wu Q., Zhang S., Song H.-F., Troyer M., Soluyanov A.A. (2018). WannierTools: An open-source software package for novel topological materials. Comput. Phys. Commun..

[57] Xu X., Ma Y., Zhang T., Lei C., Huang B., Dai Y. (2019). Nonmetal-atom-doping-induced valley polarization in single-layer Tl_2_O. J. Phys. Chem. Lett..

[58] Li L., Shao L., Liu X., Gao A., Wang H., Zheng B., Hou G., Shehzad K., Yu L., Miao F. (2020). Room-temperature valleytronic transistor. Nat. Nanotechnol..

[59] Rivera P., Seyler K.L., Yu H., Schaibley J.R., Yan J., Mandrus D.G., Yao W., Xu X. (2016). Valley-polarized exciton dynamics in a 2D semiconductor heterostructure. Science.

[60] Weiss A., John B. (1964). Goodenough: Magnetism and the chemical bond. Interscience Publishers. New York, London 1963. 393 Seiten, 89 Abbildungen. Preis: DM 95 s. Berichte Bunsenges. Phys. Chem..

[61] Cao T., Wang G., Han W., Ye H., Zhu C., Shi J., Niu Q., Tan P., Wang E., Liu B. (2012). Valley-selective circular dichroism of monolayer molybdenum disulphide. Nat. Commun..

[62] Yao W., Xiao D., Niu Q. (2008). Valley-dependent optoelectronics from inversion symmetry breaking. Phys. Rev. B.

[63] Li X., Ma X., Gao H., Zhang X., Ai H., Li W., Zhao M. (2018). Valley-selective circular dichroism and high carrier mobility of graphene-like BC_6_N. Nanoscale.

[64] Thouless D.J., Kohmoto M., Nightingale M.P., Den Nijs M. (1982). Quantized Hall conductance in a two-dimensional periodic potential. Phys. Rev. Lett..

[65] Yao Y., Kleinman L., Macdonald A.H., Sinova J., Jungwirth T., Wang D.-S., Wang E., Niu Q. (2004). First principles calculation of anomalous Hall conductivity in ferromagnetic bcc Fe. Phys. Rev. Lett..

[66] Huan H., Xue Y., Zhao B., Gao G., Bao H., Yang Z. (2021). Strain-induced half-valley metals and topological phase transitions in MBr_2_ monolayers (M = Ru, Os). Phys. Rev. B.

[67] Kong X., Li L., Liang L., Peeters F.M., Liu X.-J. (2020). The magnetic; electronic, and light-induced topological properties in two-dimensional hexagonal FeX_2_ (X = Cl, Br, I) monolayers. Appl. Phys. Lett..

[68] Zhou X., Zhang R.-W., Zhang Z., Feng W., Mokrousov Y., Yao Y. (2021). Sign-reversible valley-dependent Berry phase effects in 2D valley-half-semiconductors. NPJ Comput. Mater..

[69] Li S., Wang Q., Zhang C., Guo P., Yang S.A. (2021). Correlation-driven topological and valley states in monolayer VSi_2_P_4_. Phys. Rev. B.

[70] Xiao D., Chang M.-C., Niu Q. (2010). Berry phase effects on electronic properties. Rev. Mod. Phys..

[71] Cai T., Yang S.A., Li X., Zhang F., Shi J., Yao W., Niu Q. (2013). Magnetic control of the valley degree of freedom of massive Dirac fermions with application to transition metal dichalcogenides. Phys. Rev. B.

[72] Mahon N.S., Korolik O.V., Khenkin M.V., Arnaoutakis G.E., Galagan Y., Soriūtė V., Litvinas D., Ščajev P., Katz E.A., Mazanik A.V. (2020). Photoluminescence kinetics for monitoring photoinduced processes in perovskite solar cells. Sol. Energy.

[73] Gordon J.M., Katz E.A., Feuermann D., Albu-Yaron A., Levy M., Tenne R. (2008). Singular MoS_2_, SiO_2_ and Si nanostructures—Synthesis by solar ablation. J. Mater. Chem..

[74] You J., Pan J., Shang S.-L., Xu X., Liu Z., Li J., Liu H., Kang T., Xu M., Li S. (2022). Salt-assisted selective growth of H-phase monolayer VSe_2_ with apparent hole transport behavior. Nano Lett..

