# Supplementary material for: Ferrovalley and Quantum Anomalous Hall Effect in Janus TiTeCl Monolayer
Source: Materials (Basel). 2024 Jul 5;17(13):3331. doi: 10.3390/ma17133331 (PMC11243056; doi:10.3390/ma17133331)
Supplement: Supplementary file 1 [file materials-17-03331-s001.zip › materials-3077392-supplementary.pdf]

materials MDPI

Supplemental Material

Ferrovalley and Quantum Anomalous Hall Effect in Janus TiTeCl Monolayer

Yufang Chang [1], Zhijun Zhang [2], Li Deng [3], Yanzhao Wu [3] and Xianmin Zhang [3,*]

[1] Public Basic Department, Shenyang Conservatory of Music, Shenyang 110818, China;
[2] Liaoning Institute of Science and Technology, Benxi, 117004, China;
[3] School of Material Science and Engineering, Northeastern University, Shenyang, 110819, China;
* zhangxm@atm.neu.edu.cn

The contrasting behavior of the K and K' valleys in the VBM compared to the sizable valley polarization in the CBM in TiTeCl monolayer can be attributed to the contributions of specific orbitals to the band edges [1-3]. Since the FM ordering of TiTeCl monolayer breaks spin degeneracy between the spin-up and spin-down bands, the SOC Hamiltonian only involves the interaction of the same spin states, which can be approximately written as [1–3]:

$$\hat{H}_{Soc} \approx \hat{H}^0_{SOC} = \lambda \hat{S}_{z'} \left(\hat{L}_z \cos\theta + \frac{1}{2}\hat{L}_+ e^{-i\varnothing}\sin\theta + \frac{1}{2}\hat{L}_- e^{+i\varnothing}\sin\theta \right) \quad (S1)$$

where $\hat{L}_z$ and $\hat{S}_{z'}$ represent the z'/z components of orbital angular momentum and the spin angular momentum; (x, y, z) and (x', y', z') are the coordinate systems of $\hat{L}_z$ and $\hat{S}_{z'}$, respectively. θ is the polar angle of spin orientation (shown in inset of Figure 3d), it is zero along the +z direction.

$$\hat{L}_+ = \hat{L}_x + i\hat{L}_y,\ \hat{L}_- = \hat{L}_x - i\hat{L}_y \quad (S2)$$

The CBM is mainly contributed from d_{xy} and $d_{x^2-y^2}$ orbitals of the Ti atom, while the VBM is mainly contributed from d_{z^2} of the Ti atom, as descript in Figures S1(a)-S1(c). Comparatively, there is little distribution of the d_{xz} and d_{yz} orbitals of Ti atom near the valley, as drawn in Figures S1(d) and S1(e). Since the symmetry group of the K and K′ points are D_{3h}, accordingly, the basic functions are $\left|\psi_c^\tau\right\rangle = \frac{1}{\sqrt{2}}(\left|d_{x^2-y^2}\right\rangle + i\tau\left|d_{xy}\right\rangle)$, $\left|\psi_v^\tau\right\rangle = \left|d_{z^2}\right\rangle$, where $\tau = \pm 1$ refers to the valley index and c/v refers to the conduction and valence bands. Therefore, the energy levels of the valleys at CBM and VBM are described by:

$$E_c^\tau = \langle\psi_c^\tau|\hat{H}^0_{SOC}|\psi_c^\tau\rangle,\ E_v^\tau = \langle\psi_v^\tau|\hat{H}^0_{SOC}|\psi_v^\tau\rangle \quad (S3)$$

The valley polarizations could be given by:

$$E_c^+ - E_c^- = i\langle d_{x^2-y^2}|\hat{H}^0_{SOC}|d_{xy}\rangle - i\langle d_{xy}|\hat{H}^0_{SOC}|d_{x^2-y^2}\rangle \quad (S4)$$

$$E_v^+ - E_v^- \approx 0 \quad (S5)$$

The basic functions $\left|d_{x^2-y^2}\right\rangle$ and $\left|d_{xy}\right\rangle$ could be given by:

$$\left|d_{x^2-y^2}\right\rangle = \frac{1}{\sqrt{2}}(|d_{+2}\rangle + |d_{-2}\rangle) \quad (S6)$$

$$\left|d_{xy}\right\rangle = \frac{1}{\sqrt{2}}[-i(|d_{+2}\rangle - |d_{-2}\rangle)] \quad (S7)$$

Thus, one can obtain $E_c^+ - E_c^- \propto 4\cos\theta$, which means that the valley polarization will vary as the cosine function of the polar angles of spin orientation. This is consistent with the calculated results in Figure 3(d).

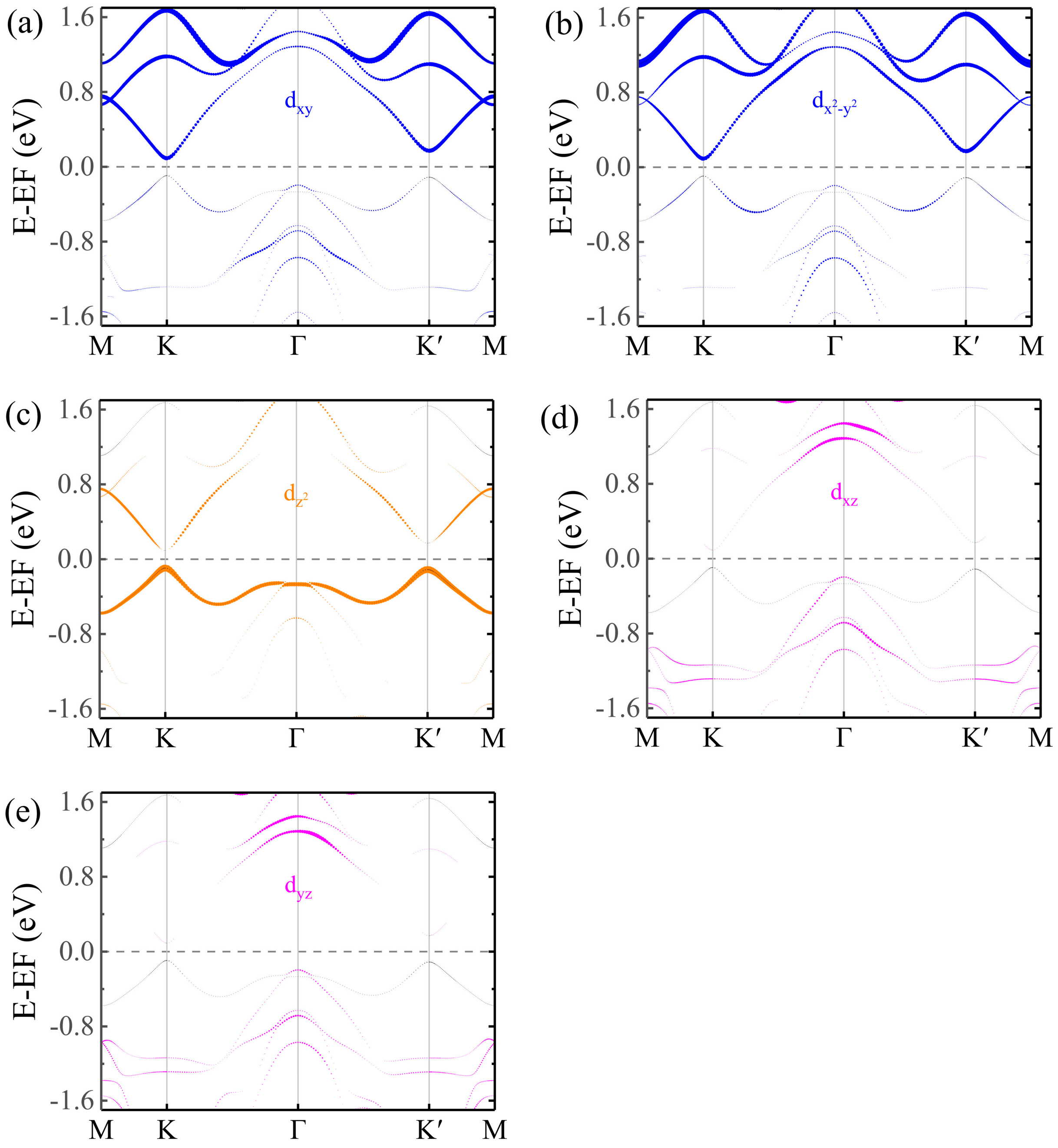

Figure S1. Projected band structures of Ti-d orbitals for TiTeCl monolayer.

References

1. R. Li, J. Jiang, W. Mi, H. Bai, Room temperature spontaneous valley polarization in two-dimensional FeClBr monolayer, *Nanoscale* **13**, 14807 (2021).
2. W.-Y. Tong, S.-J. Gong, X. Wan, and C.-G. Duan, Concepts of ferrovalley material and anomalous valley Hall effect, *Nature Communications* **7**, 13612 (2016).
3. Q. Cui, Y. Zhu, J. Liang, P. Cui, H. Yang, Spin-valley coupling in a two-dimensional VSi2N4 monolayer, *Physical Review B* **103**, 085421 (2021).
